# Fronto-orbital advancement with patient-specific 3D-printed implants and robot-guided laser osteotomy: an in vitro accuracy assessment

**DOI:** 10.1007/s11548-024-03298-6

**Published:** 2024-12-13

**Authors:** Michaela Maintz, Nora Desan, Neha Sharma, Jörg Beinemann, Michel Beyer, Daniel Seiler, Philipp Honigmann, Jehuda Soleman, Raphael Guzman, Philippe C. Cattin, Florian M. Thieringer

**Affiliations:** 1 Medical Additive Manufacturing Research Group (Swiss MAM), Department of Biomedical Engineering, University of Basel, Allschwil, Switzerland; 2 Institute for Medical Engineering and Medical Informatics, University of Applied Sciences and Arts Northwestern Switzerland, Muttenz, Switzerland; 3 Clinic Oral and Cranio-Maxillofacial Surgery, University Hospital Basel, Basel, Switzerland; 4 Hand- and Peripheral Nerve Surgery, Department of Orthopaedic Surgery and Traumatology, Kantonsspital Baselland, Bruderholz Liestal Laufen, Switzerland; 5 Biomedical Engineering and Physics, Amsterdam UMC Location University of Amsterdam, Amsterdam, The Netherlands; 6 Department of Neurosurgery, University Hospital Basel, Basel, Switzerland; 7 Division of Pediatric Neurosurgery, Children’s University Hospital Basel, Basel, Switzerland; 8 Center of Medical Image Analysis and Navigation (CIAN), Department of Biomedical Engineering, University of Basel, Allschwil, Basel, Switzerland; 9 Faculty of Medicine, University of Basel, Basel, Switzerland

**Keywords:** Patient-specific implants, Robot-assisted surgery, Laser osteotome, Pediatrics, Computer-aided design, Craniosynostosis

## Abstract

**Purpose:**

The use of computer-assisted virtual surgical planning (VSP) for craniosynostosis surgery is gaining increasing implementation in the clinics. However, accurately transferring the preoperative planning data to the operating room remains challenging. We introduced and investigated a fully digital workflow to perform fronto-orbital advancement (FOA) surgery using 3D-printed patient-specific implants (PSIs) and cold-ablation robot-guided laser osteotomy. This novel approach eliminates the need for traditional surgical templates while enhancing precision and customization, offering a more streamlined and efficient surgical process.

**Methods:**

Computed tomography data of a patient with craniosynostosis were used to digitally reconstruct the skull and to perform VSP of the FOA. In total, six PSIs per skull were 3D-printed with a medical-grade bioresorbable composite using the Arburg Plastic Freeforming technology. The planned osteotomy paths and the screw holes, including their positions and axis angles, were digitally transferred to the cold-ablation robot-guided osteotome interface. The osteotomies were performed on 3D-printed patient skull models. The implants, osteotomy and final FOA results were scanned and compared to the VSP data.

**Results:**

The osteotomy deviations for the skulls indicated an overall maximum distance of 1.7 mm, a median deviation of 0.44 mm, and a maximum root mean square (RMS) error of 0.67 mm. The deviation of the point-to-point surface comparison of the FOA with the VSP data resulted in a median accuracy of 1.27 mm. Accessing the orbital cavity with the laser remained challenging.

**Conclusion:**

This in vitro study showcases a novel FOA technique by effectively combining robot-guided laser osteotomy with 3D-printed patient-specific implants, eliminating the need for surgical templates and achieving high accuracy in bone cutting and positioning. The workflow holds promise for reducing preoperative planning time and increasing surgical efficiency. Further studies on bone tissue are required to validate the safety and effectiveness of this approach, especially in addressing the challenges of pediatric craniofacial surgery.

## Introduction

Fronto-orbital advancement (FOA) is a reconstructive surgical technique for treating metopic and coronal craniosynostosis. This congenital disorder is characterized by the premature fusion of cranial sutures, affecting about 5 per 10,000 live births [1–3], with rising prevalence [4]. This condition leads to morphological skull deformities with potential elevated intracranial pressure and psychosocial issues [3, 5]. Despite significant advances in craniosynostosis management, surgery is still the preferred treatment in the majority of cases.

Although minimally invasive endoscopic procedures have been proposed [6], these are typically employed in infants under the age of four months, while helmet therapy is recommended for infants between 6 and 12 months. The most widely acknowledged treatment to restore the calvarial shape includes open cranial vault remodeling. Nowadays, the surgical correction of craniosynostosis relies heavily on the surgeon's subjective judgment in assessing the degree of the deformity and the best approach to remodel the affected bone to restore a normal skull shape. This method usually lengthens the surgery and depends highly on the surgeon's experience.

Technological advancements in computer-aided design (CAD), computer-assisted manufacturing (CAM) [7, 8] and surgical navigation [9, 10] have significantly improved the accuracy and efficiency of computer-assisted surgical planning. Augmented reality (AR) has emerged as a novel tool in surgical guidance, providing enhanced visualization by overlaying virtual models onto the patient’s anatomy. Han et al. (2019) describe a new method for cranial vault reconstruction using augmented reality in synostotic plagiocephaly surgery, while García-Mato et al. (2020) further support the submillimetric precision of AR in guiding both osteotomies and bone remodeling procedures [11, 12].

In a first step, usually the patient’s bone structures, derived from computed tomography (CT) scans, are visualized in three dimensions and compared with normative models for more objective virtual surgical planning (VSP) [13]. Additionally, 3D-printed templates help transfer these plans to surgery, facilitating placement of reshaped bone segments [10, 14, 15]. Although this method seems to be the current gold standard, the surgical planning procedure, including the 3D printing process of the surgical templates, remains a time-consuming task. At least two templates must be produced which guide the cutting and positioning of the bone segments [14–17]. An accurate fit of the surgical guide to the skull is not guaranteed, and minor errors in bone placement can compromise symmetry. Reducing direct contact with bone improves sterility and lowers infection risks. A robot-guided laser can perform contactless osteotomies, minimizing thermal and mechanical damage to the tissue [18]. While intraoperative optical tracking systems and AR provide real-time guidance, they still rely on manual precision, introducing variability. Robot-guided lasers offer highly precise cuts, crucial for achieving accurate, symmetrical outcomes in craniosynostosis surgeries.

In this context, robot-guided surgery can assist the osteotomies by pre-drilling the screw holes during the bone separating. Robotic-guided instruments possess the ability to transfer pre-operative plans to the patient in the operating room with high precision and have been used in many fields to improve the performance and safety of surgical procedures [19]. Clinical trials using robot-guided laser midface osteotomies have shown promising results in orthognathic surgery applications [20]. To our knowledge, this promising technology has not yet been applied or evaluated in craniosynostosis surgery. When combined with 3D-printing, it enables the fabrication of patient-specific bioresorbable plates that precisely guide and fix bone segments according to pre-surgical plans. Using 3D-printed implants results in accurate and efficient surgical outcomes for complex bone rearrangements, reducing manual adjustments and reliance on prefabricated templates [8].

In this in vitro study, we introduce a novel digital workflow for craniosynostosis surgical correction, utilizing a cold-ablation robot-guided laser osteotome and 3D-printed bioresorbable patient-specific implants (PSIs). Our objective was to establish a digital workflow and evaluate the translational accuracy of the VSP for osteotomies and positioning of remodeled bone segments using these technologies in craniosynostosis surgical procedures.

## Materials and methods

### Study protocol and ethical considerations

**Fig. 1:**
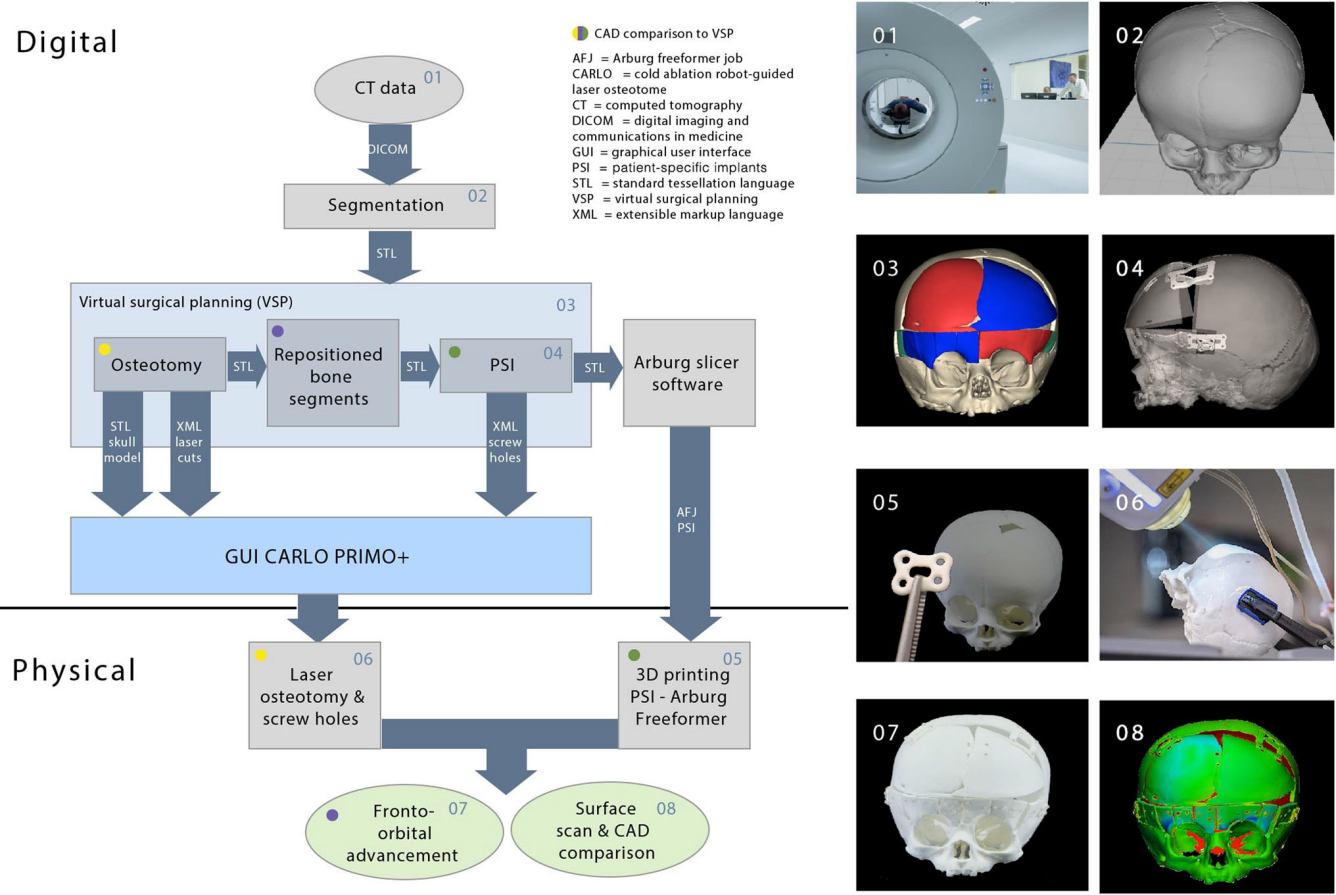
The study's workflow included the following steps: **1** CT scan of the patient's skull. **2** Skull segmentation. **3** Virtual surgical planning (VSP) including **4** design of patient-specific implants (PSIs). **5** 3D-printing of the skull and PSIs (*n* = 3). **6** Robot-guided laser osteotomy and screw hole pre-drilling. **7** Reconstruction of bone segments with PSIs in a simplified fronto-orbital advancement (FOA), assisted by the laser osteotome. **8** 3D scanning and accuracy analysis of the repositioned skull segments

Our study involved digital and physical processes to design and create patient-specific 3D skull models and bioresorbable implants for osteotomies and repositioning. We utilized a cold-ablation robot-guided laser system to perform the osteotomies according to the VSP, while optical surface scanning was applied for data analysis and evaluation, as outlined in Fig. 1.

The study was conducted in full compliance with the pertinent guidelines and regulations that govern medical research, in concordance with the Swiss Federal Act on Data Protection. An anonymized CT Digital Imaging and Communications in Medicine (DICOM) dataset was utilized in this study, obtained from the comprehensive database of the University Hospital Basel. This was a non-clinical, in silico, experimental study encompassing an anonymized dataset from a patient who had undergone FOA procedures at the University Hospital Basel. Due to the study's non-clinical nature and exploratory objectives, coupled with the fact that simulations were exclusively conducted on anonymized virtual models without any translational impact on clinical practice, the study did not necessitate ethical approval as per the Swiss Association of Research Ethics Committees [21].

## Virtual surgical planning and 3D-printing

**Fig. 2:**
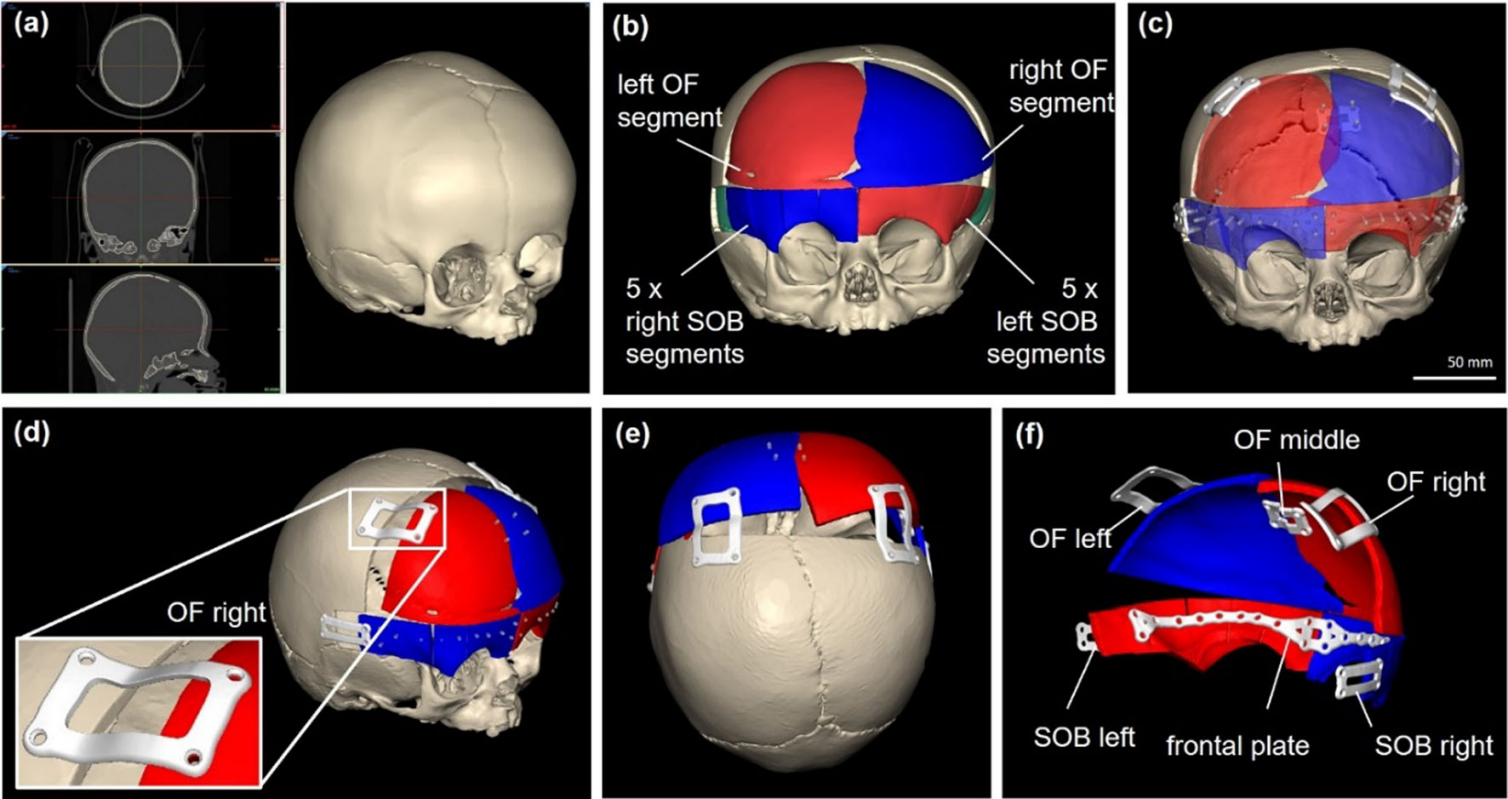
**a** 3D reconstruction of CT data for a craniosynostosis patient's skull. **b** Illustration of osteotomy and advancement of left and right frontal (Os frontale—OF) and supra-orbital bandeau (SOB) segments. **c** Design of patient-specific implants (PSIs) for fixing OF and SOB. **d** Right-lateral view and detailed image of the plate for fixing the right frontal bone (OF right). **e** Top view of the skull showing advanced bone segments and plates. **f** Posterior-lateral view of bone segments and plate labels

The CT DICOM dataset was imported into Mimics Medical (v.24.0, Materialise, Leuven, Belgium) with the following image parameters 0.375 × 0.375 mm^2^ pixel spacing and 0.6 mm slice thickness. The image data were segmented (Fig. 2a) and the VSP procedure incorporated the repositioning of the Os frontale (OF) and supraorbital bandeau (SOB) segments. The VSP involved the separation of the frontal bone along the metopic and coronal sutures. The fragments were repositioned and advanced (Fig. 2b, c) using normative reference skull models [15]. The 3D models of the original and remodeled skull were exported in Standard Tessellation Language (STL) file format.

PSIs were designed using Geomagic Freeform Plus (v. 2019, 3D Systems, Rock Hill, USA), importing STL files of skull models. Six osteosynthesis plates, each 1.5 mm thick and 4.0 mm wide with 42 screw axis markers, were created to stabilize the ten SOB segments (Fig. 2 d-f) and connect the advanced supraorbital bony segments to the intact temporal bones on both sides (SOB left, SOB right). All PSI STL files were exported for manufacturing.

**Fig. 3.**
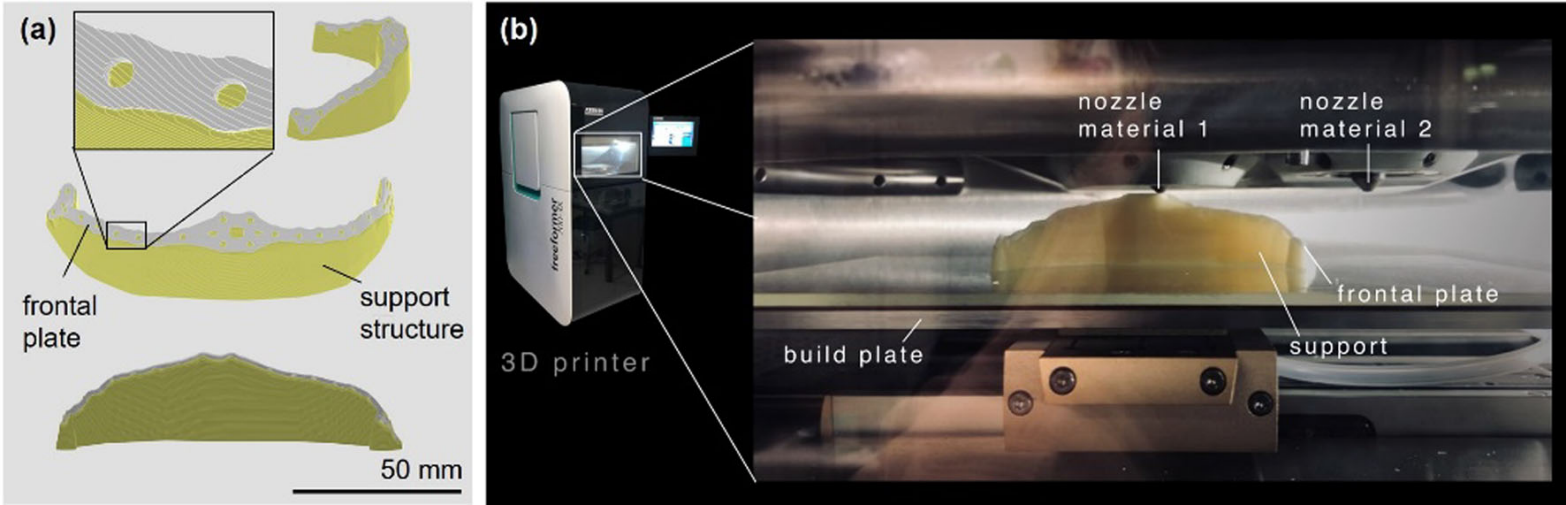
3D printing of the frontal plate. **a** Side, front, and back views of the frontal plate in slicing software, displaying the support structure and a magnified view of the slice layers of the supports and the plate. **b** View of the Arburg Freeformer 200-3X build chamber during 3D printing of the frontal plate, using poly(L-lactide-co-D,L-lactide) with 30% β-TCP for nozzle material 1 and Armat11 (a water-soluble support) for nozzle material 2

The implant’s STL data were oriented and processed in the slicing software, Arburg Freeformer (v. 2.31, Arburg GmbH + Co KH, Lossburg, Germany), generating necessary support structures (Fig. 3). Skull models and PSIs were 3D-printed from polyamide 12 and RESOMER LR 706 S β-TCP (30% β-tricalcium phosphate, poly(L-lactide-co-D,L-lactide), Evonik Industries AG, Essen, Germany) using Selective Laser Sintering and Arburg Plastic Freeforming (APF), respectively. 3D printing of the implant involved using APF technology to precisely deposit layers of medical-grade bioresorbable material, creating detailed structures as per the digitally designed specifications. Post-processing included dissolving the support material in lukewarm water (35–38 °C) and manual drilling of screw holes to ensure proper screw fit.

## Cold-ablation robot-guided laser osteotomy

**Fig. 4:**
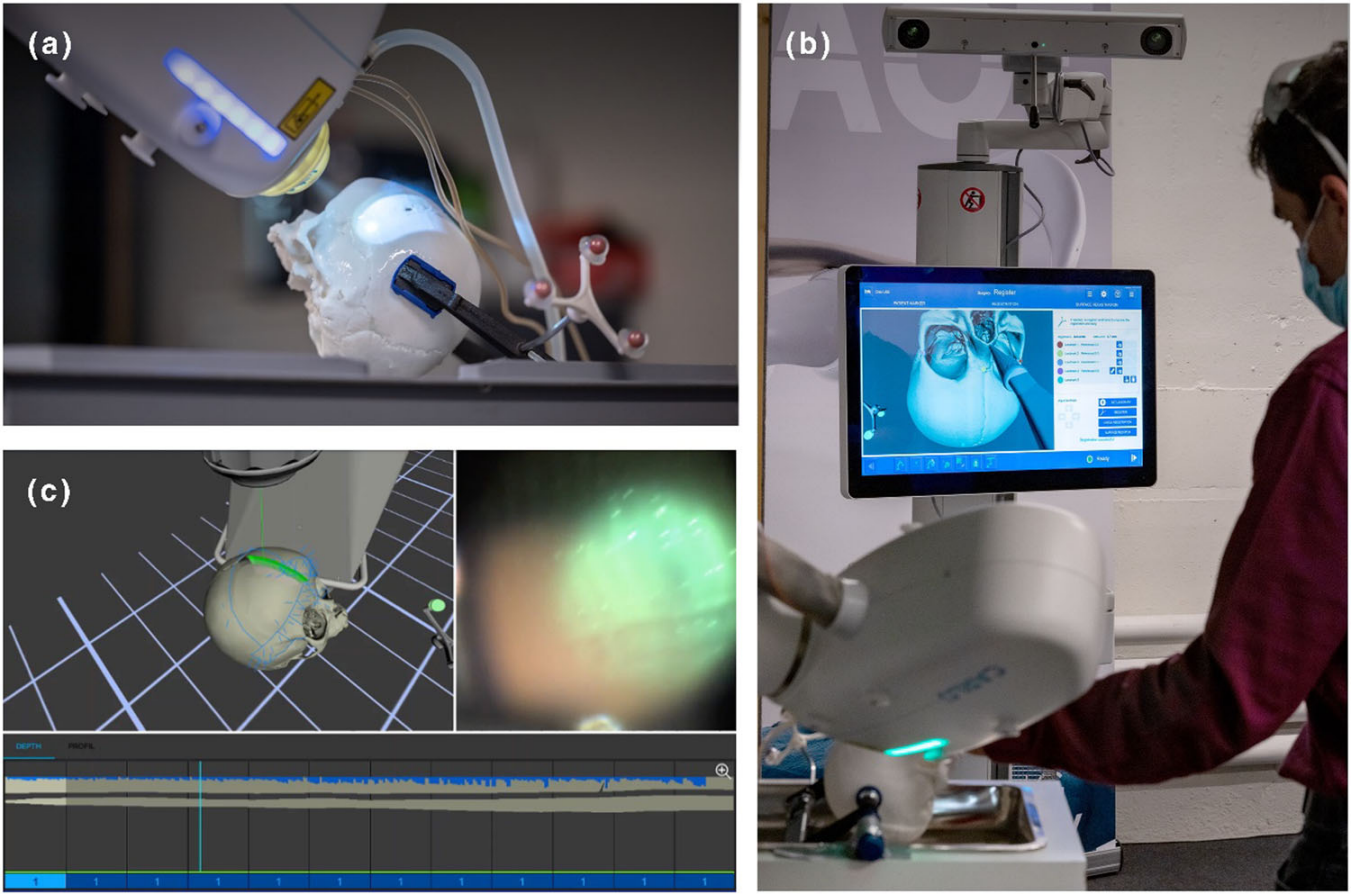
Laser osteotomy of a 3D-printed plastic skull model based on virtual surgical planning (VSP) data with the cold-ablation robot-guided laser osteotome. **a** Skull fixated on operation table with reference marker. **b** View of the user interface during the registration process. **c** Graphical user interface (GUI): View of the skull in 3D space with the pre-operatively planned osteotomy planes, camera view of the bone surface and view of depth control using optical coherence tomography (OCT)

Osteotomy and screw hole paths from VSP data were exported as XML files and transferred to the CARLO® primo + interface. Defined trajectories for each hole, including entry points and angles, were imported directly into the cold-ablation robot-guided laser osteotome (CARLO® primo + , Advanced Osteotomy Tools AOT AG, Basel, Switzerland) graphical user interface (GUI), creating precise cutting paths without manual adjustments (Fig. 4).

### Osteotomy

The laser osteotome CARLO® primo +  uses a 2.94 μm Erbium-doped Yttrium Aluminum Garnet laser [18, 22, 23]. This device executes precise cuts through a series of aligned pulses, segmented and monitored using optical coherence tomography (OCT) and a coaxial camera system. It begins by creating a 1-mm wide and deep groove, with each subsequent pulse progressively deepening the cut until the bone segments are completely separated. Skull 0 underwent a complete separation with three pulses to fine-tune the workflow parameters, whereas Skulls A and B received an incomplete separation with just two pulses. Additionally, the osteotome generated 42 screw holes per skull using precise single-point shots, with the OCT system continuously measuring the depth.

## Fronto-orbital advancement

**Fig. 5:**
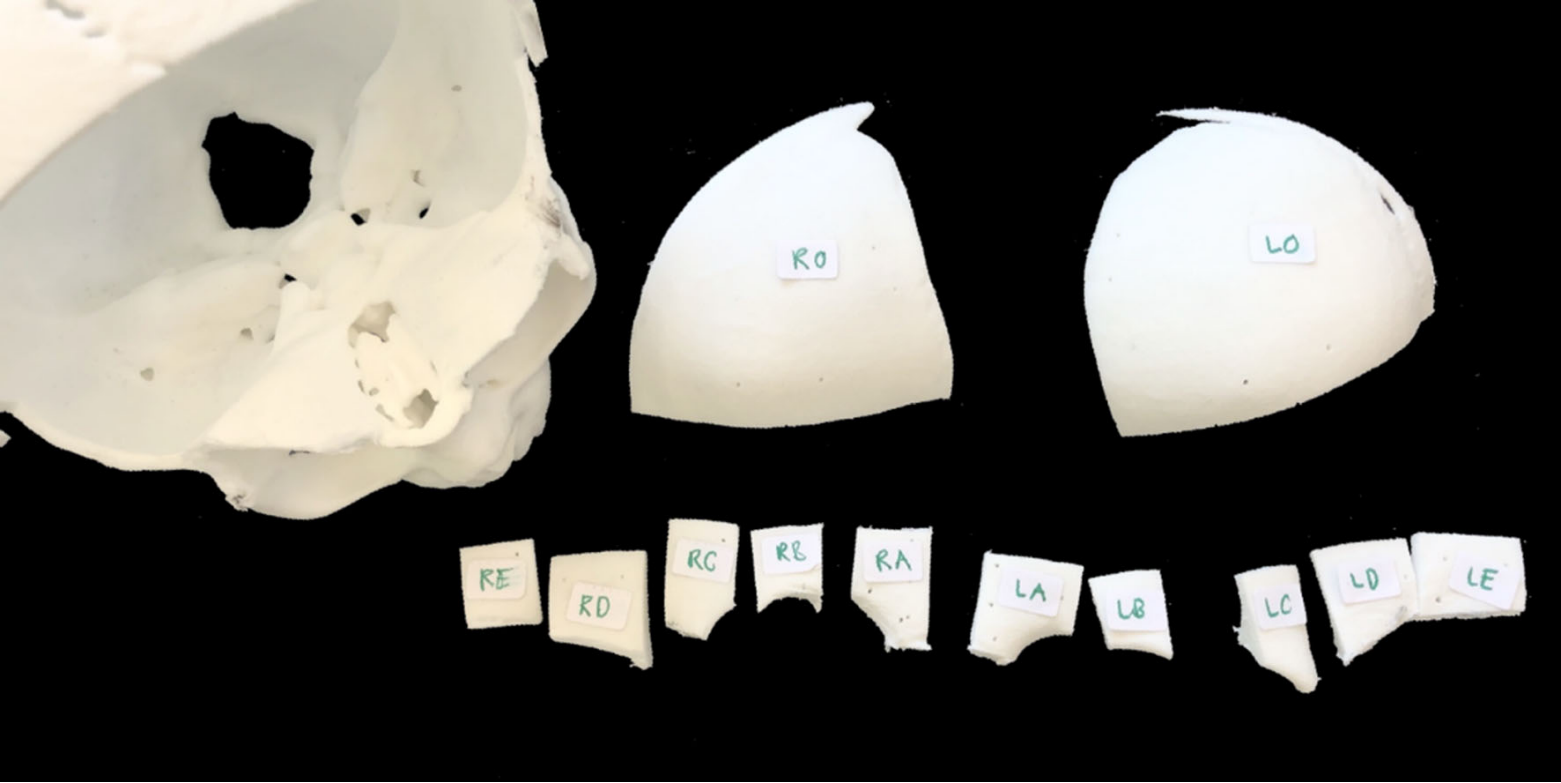
Depiction of labeled bony segments (RO—right Os frontale, LO—left Os frontale, RA-RE—segments of the right supraorbital bandeau, LA-LE—segments of the left supraorbital bandeau)

The CARLO® primo +  conducted the initial osteotomy, but manual dissection was required for unreachable orbital areas. Incomplete osteotomies in Skulls A and B, detected through 3D scans, led to manual separation before securing the bone segments with PSIs and 2.0 mm screws (Fig. 5). The implants were positioned based on the laser-created holes, with the reconstruction involving reattachment from the orbital rim to the upper frontoparietal regions, stabilizing the structure without additional support.

## Accuracy assessment

The surface shape deviation for two intact skull models (Skull A and B) and six implants (*n* = 3) assessed using the 3D optical scanner (Transcan C, Shining 3D, v. 1.4.2.3, Hangzhou, China). Scans were compared to original STL files using medically certified CAD software (3-matic Medical, v. 18, Materialise, Leuven, Belgium) through N-point and global registration, aligning scanned parts for analysis. Discrepancies were measured using the “Part Comparison Analysis” function, focusing on skull areas near the planned FOA. The final surface-to-surface distances (median and root mean square (RMS)) were computed and compared based on the absolute distance values.

Implant thicknesses were measured at five random points on the implant surface scans using Materialise 3-matic software. Skull models (Skull A and B) with osteotomy grooves were scanned and aligned to the VSP data before advancing bone segments. Point-to-point distance was evaluated by placing thirty equidistant points along the grooves to measure deviations from the planned cutting path, calculating median Euclidian and root-mean-square deviations RMS distances to assess osteotomy accuracy. FOA accuracy was assessed by measuring point-to-point Euclidian distances between 46 points on corner nodes of bone segments from the optical scans of the FOA models (Skull 0, Skull A, Skull B) and their VSP coordinates. During registration of the FOA model scans to the VSP model, the N-point method was by marking landmarks on the non-osteotomized bone, without applying global registration. In three steps, 10 points were, respectively, manually selected on each skull, to align the 3D models. This approach ensured precise alignment with the skull and minimized registration errors that could arise from inaccurate positioning of the bony segments.

## Results

### 3D-printed skull models and implants

**Fig. 6:**
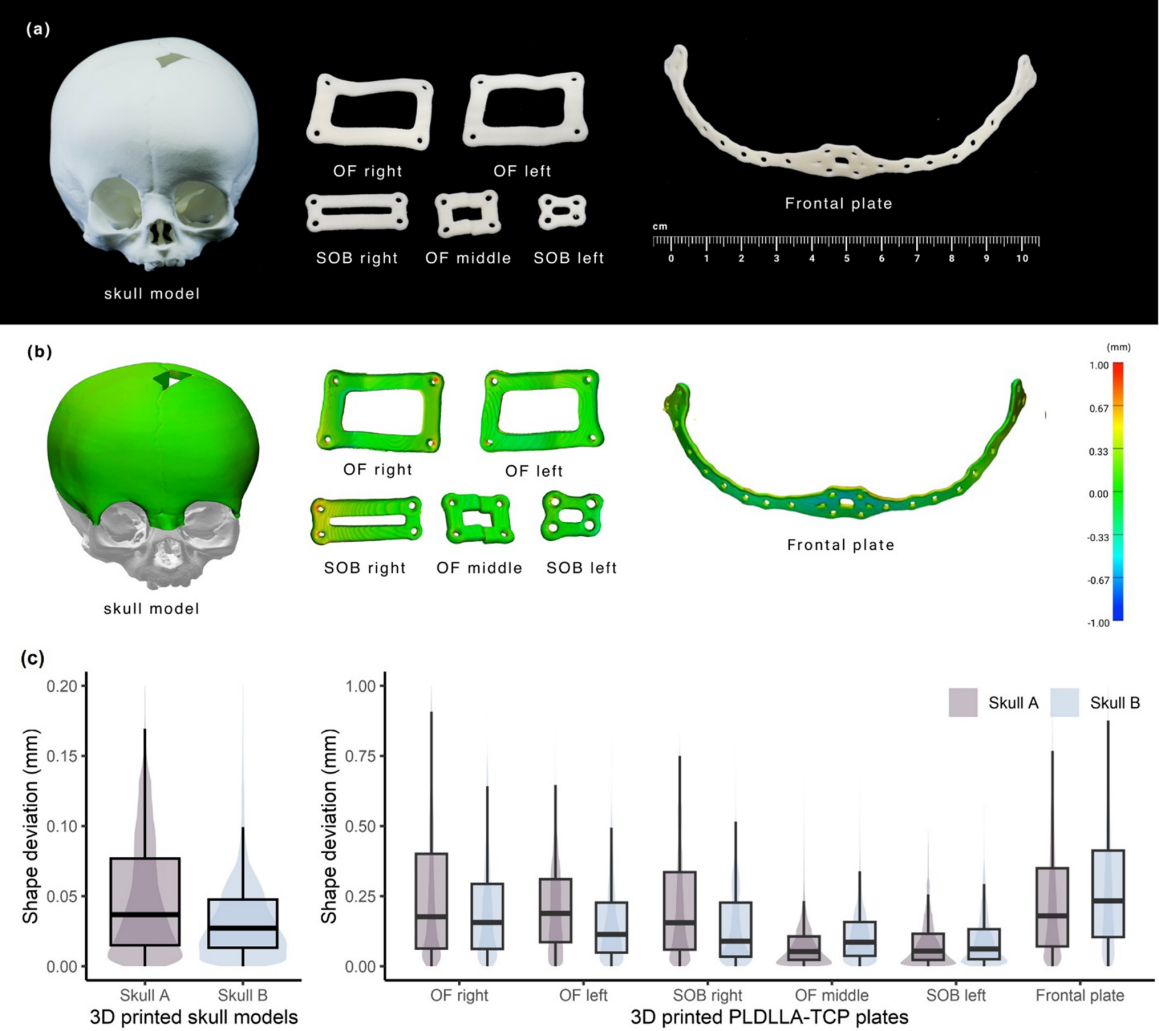
**a** Physical prototypes of 3D-printed skull model and 3D-printed poly(L-lactide-co-D,L-lactide) with β-TCP (PLDLLA/TCP) plates. **b** Color-coded map depicting the surface distances of the 3D-printed skull model and implant surface scan to the respective CAD models (for Skull A). **c** Box- and violin plots of the surface deviation of the 3D-printed skull model and the implants to the CAD models

The surface comparison results of Skull A and Skull B models and the implants are listed in Table 1. The 3D-printed intact skulls were compared with the segmented skull of the patient’s CT scan. As shown in Fig. 6b, artifacts caused by the limited accessibility of the 3D scanner restricted the analysis to only the area of the skull near the planned FOA. On average, the surface comparison of Skull A and Skull B to the initial 3D geometry showed a median deviation of 0.03–0.04 mm.

**Table 1:** Summary of the assessed objects, methods used, and corresponding samples (FOA (fronto-orbital advancement))

	Assessed object	Method	Sample
1	Intact skull models and implants	3D surface deviation analysis	Skull A, Skull B (intact before osteotomy) and six implants for FOA
2	Implant thickness	Point-to-point distance measurement	Six implants for FOA
3	Osteotomy	Point-to-point deviation analysis	Skull A and Skull B (with laser osteotomy)
4	FOA	Point-to-point deviation analysis	Skull 0, Skull A and Skull B (with FOA)

The 3D-printed implants were compared to the original CAD geometry. Figure 6a depicts all six 3D-printed implants. In each case, the deviations for the different implants are visualized in Fig. 6b. Overall, the 3D-printed PSI surfaces show a median deviation of 0.11 mm and RMS of 0.23 mm (Fig. 6c). The mean thickness of all implants was 1.52 ± 0.09 mm after 3D printing.

## Osteotomy

**Fig. 7:**
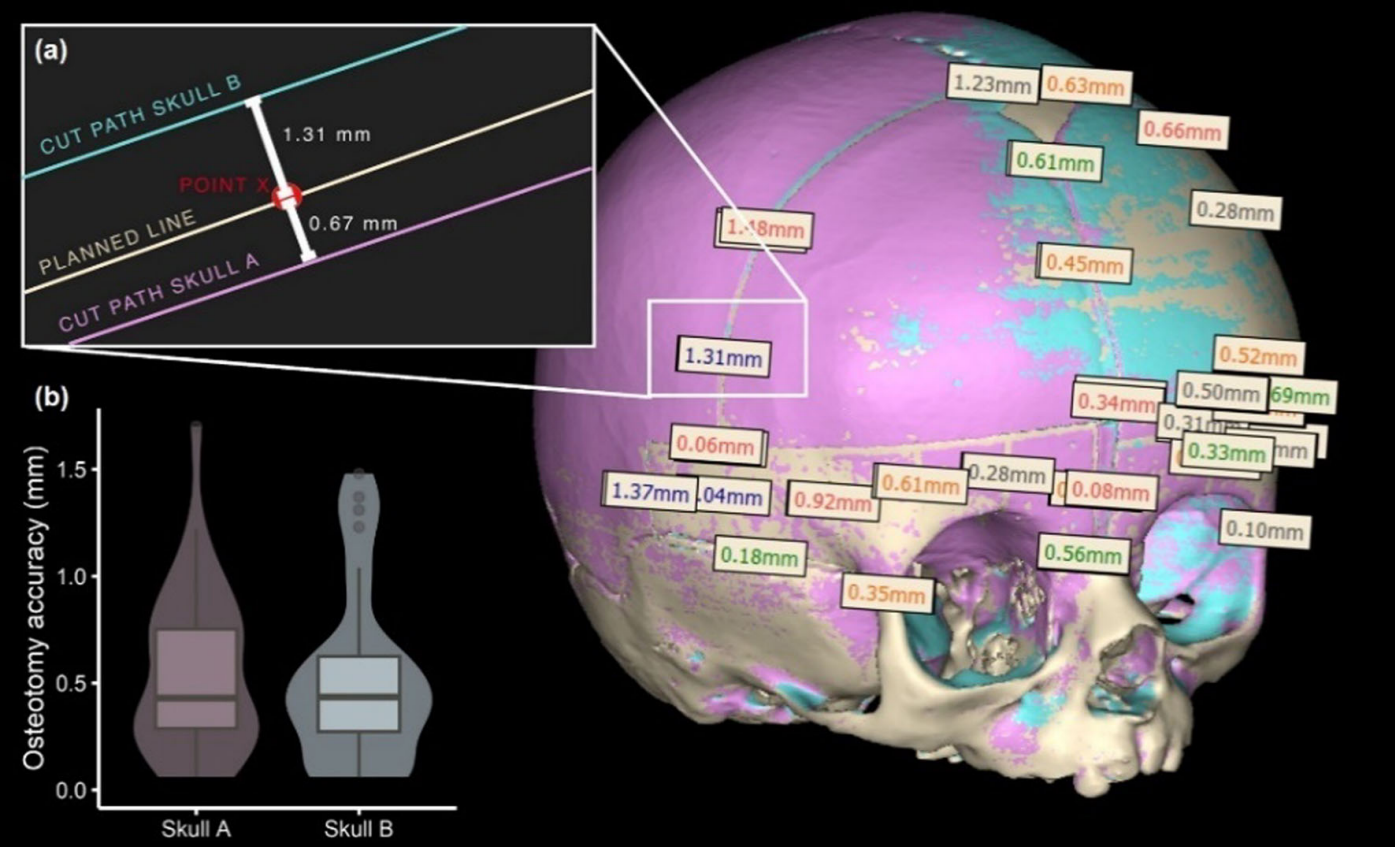
Measurement of the osteotomy accuracy: superposition of 3D scans of Skull A (purple) and Skull B (blue) on the virtual planning data of skull (beige) with osteotomy. **a** Close-up view of the osteotomy lines (cut paths) of Skull A and Skull B and exemplary distance measurement to point x, located on the virtually planned osteotomy line. **b** Box- and violin plots of the point-to-point osteotomy measurements

The osteotomies of Skull A and Skull B took approximately three hours. The point-to-point distance values of the 30 points of the scanned osteotomy lines (Skull A and Skull B) to the planned cuts are listed in Table 2. An overview of the measuring method and results is shown in Fig. 7. The measurements of the osteotomy deviations of both skulls revealed an overall maximum distance of 1.7 mm, a median deviation of 0.44 mm and a maximum RMS error of 0.67 mm.

**Table 2:** Geometric accuracy from absolute deviations of 3D-printed skulls and implants mounted on Skull A/Skull B

	Median* (mm)	RMS* (mm)
Skull without implants	0.04	0.11
OF right	0.17	0.30
OF left	0.15	0.23
SOB right	0.13	0.24
OF middle	0.09	0.12
SOB left	0.06	0.12
Frontal plate	0.20	0.32

*Mean of both skulls and implants for Skull A and Skull B

## Fronto-orbital advancement

**Fig. 8:**
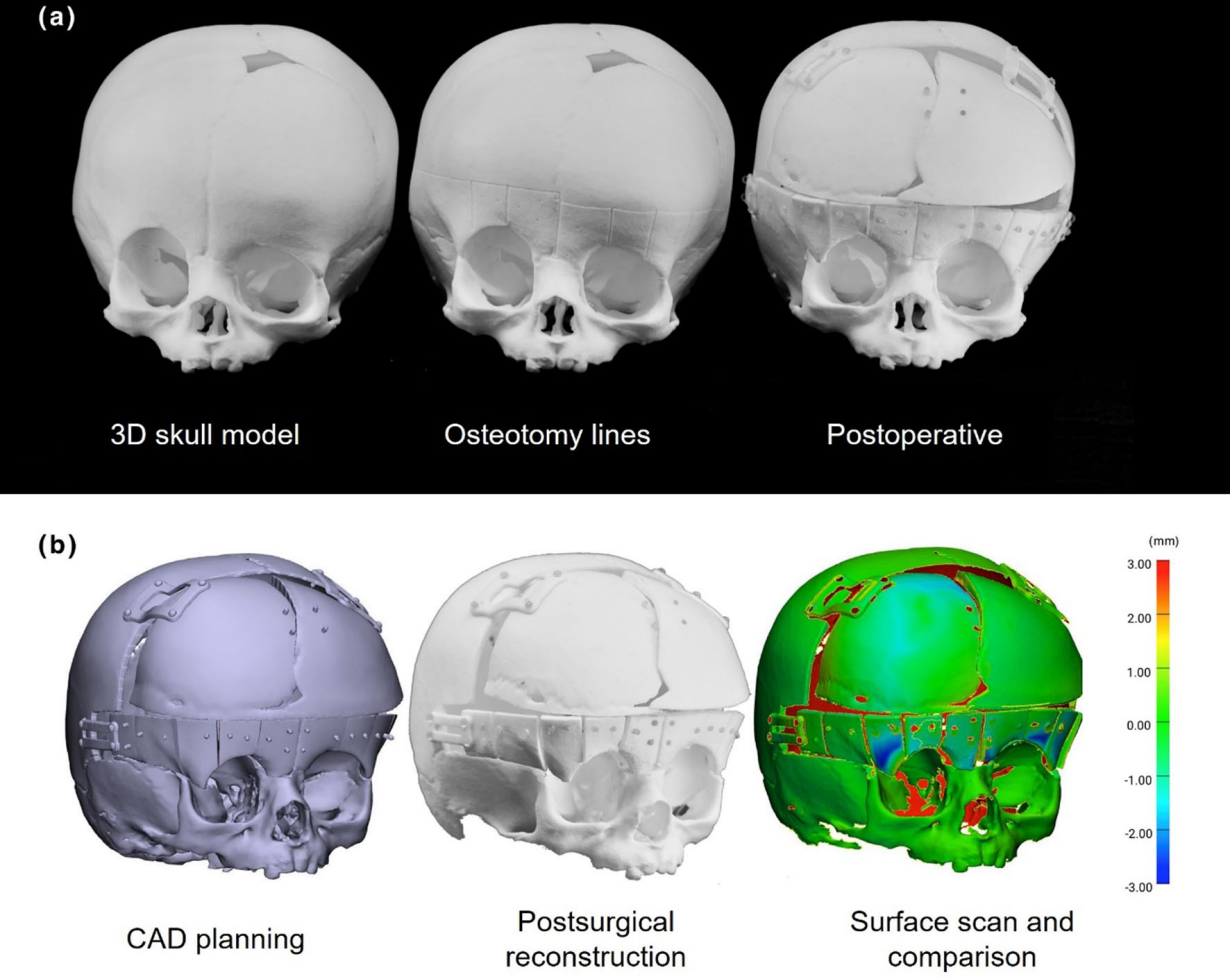
Physical and digital skull models. From left to right: **a** Intact and modified skull models showing osteotomy lines, screw holes, and repositioned segments with patient-specific implants (PSIs). **b** The resulting procedure, using computer-aided design (CAD) for the 3D-printed fronto-orbital advancement model, including a surface heatmap comparing the assembled model (Skull A) to the virtual plan (scale in mm)

**Fig. 9:**
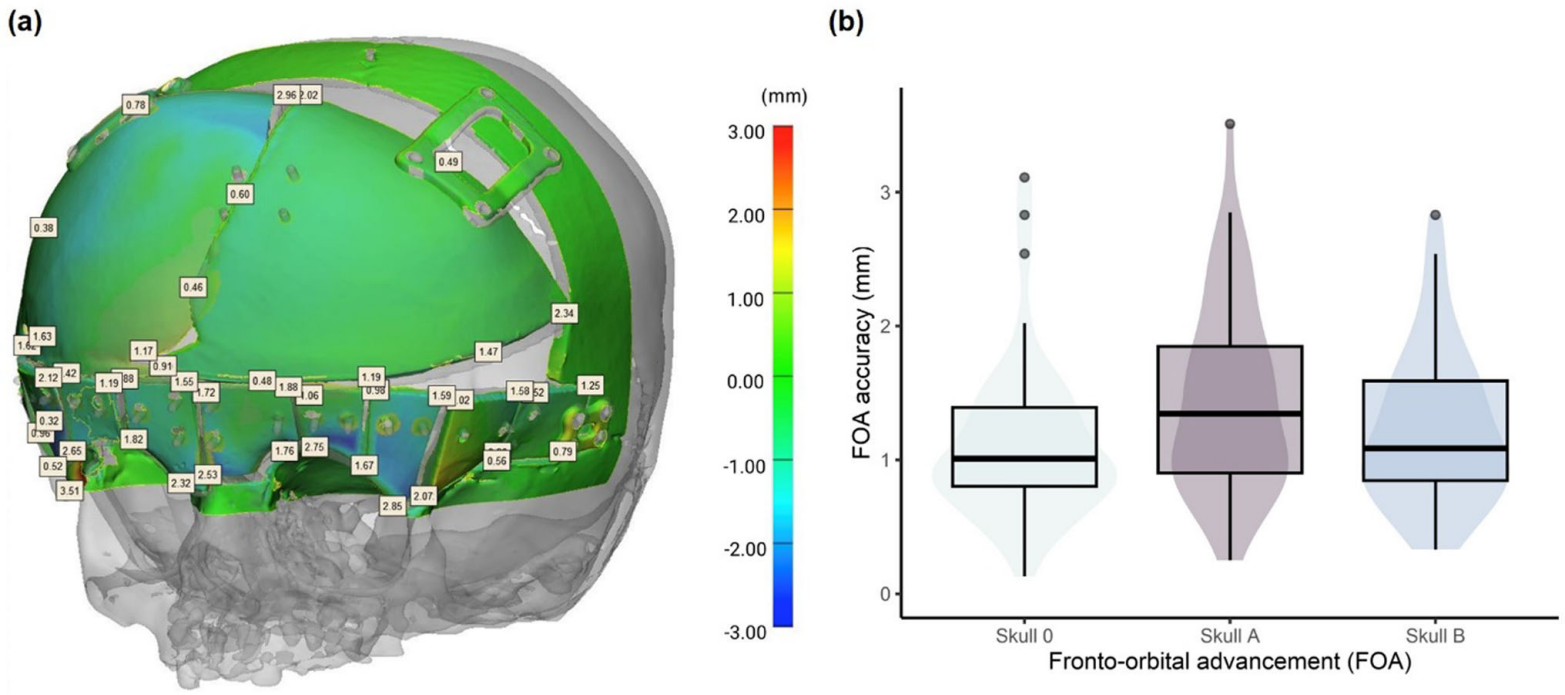
**a** Representative depiction of surface accuracy assessment and point-to-point distances of the advanced bone segments for the surface scan of Skull A (in (mm)). **b** Box- and violin plots of the manual accuracy measurement (depicted in (a)) of the 3D scanned skull segments to the virtually planned position

Figure 8a illustrates the steps to create the FOA skull model. The resulting surface scan of the skull, including the PSIs, with the positioned bone segments, was compared to the STL file of the FOA virtual planning geometry. The maximum median deviation of the three skulls (Table 4) was 0.42 mm. At the supraorbital rim, lateral to the orbital cavities, the maximum deviation of approx. 3.51 mm was reached, which is also visible in the blue areas in the heat map of the surface comparison results shown in Figs. 8b and 9a. The point-to-point distance results of the outer corners of repositioned bone segments to the virtual planning data are listed in Table 3. Additionally, the values are depicted as box- and violin plots in Fig. 9b. The median point-to-point distances among all FOA skulls are 1.27 mm. The highest median point-to-point distance was measured on Skull A, 1.35 mm and a maximum point-to-point (Hausdorff) distance of 3.51 mm was measured lateral to the right orbital cavity (Table 4).

**Table 3:** Accuracy of osteotomy measured by deviation from cut path

	Median (mm)	RMS (mm)	Max. deviation (mm)
Skull A	0.43	0.67	1.70
Skull B	0.44	0.66	1.48

**Table 4:** Surface comparison and manual measurement results of assembled, osteotomized skull with patient-specific implants

	Surface shape deviation		Point-to-point distance		
	Median (mm)	RMS (mm)	Median (mm)	RMS (mm)	Maximum distance (mm)
Skull 0	0.14	0.46	1.01	1.27	3.11
Skull A	0.42	0.79	1.35	1.62	3.51
Skull B	0.21	0.55	1.09	1.35	2.83

## Discussion

FOA is one of the most prevalent surgical interventions for addressing both syndromic and non-syndromic craniosynostosis. These procedures rely on surgeons' skills and judgment. Consequently, innovations are being developed to enhance the surgical workflow. One existing method involves creating personalized resorbable osteosynthesis plates to reduce intraoperative adjustments [8]. Clinically, resorbable plates have shown good outcomes and benefits in pediatric craniofacial surgery [24, 25] and their use inside the skull’s cortex is a safe and effective method, avoiding plate contour prominence [25].

Another approach that optimizes the osteotomy procedure is through VSP and 3D-printed patient-specific surgical cutting guides, producing reproducible results for FOA surgery [15]. Nonetheless, next to the time-consuming process of surgical template design, a perfect fit of the guide could be difficult to ensure. Problems in geometric fit could arise from guide design errors, manufacturing inaccuracies, or soft tissue obstruction. Therefore, robot-guided laser osteotomy has emerged as another precise method. The CARLO® system’s performance has been investigated in multiple studies, showing its potential as a precise tool to replace conventional instruments for bone surgery [19, 20, 26, 27]. This study assessed FOA accuracy using resorbable PSIs and robot-guided laser osteotomy for FOA in a complete digital workflow.

Implant production at the point-of-care allows flexibility for last-minute changes. This study was conducted at the University Hospital Basel's 3D Print Lab using the Arburg Freeformer 200-3X, a printer for research on biodegradable implant fabrication [28]. Printing six implants for one skull took about 3.5 h. Post-processing involved dissolving support material and drilling screw holes. Surface scan analysis of the 3D-printed PSIs showed minor deviations from the intended designs, with surface deviations ranging from 0.06 to 0.20 mm (Table 2). The largest deviation was in the frontal plate, potentially due to thermal expansion or warping during printing. Using APF technology and pre-drilled screw holes by CARLO®, the 3D-printed implants facilitate precise bony fragment advancement in surgery, eliminating the need for custom positioning templates. Another advantage is the ability to create highly customized implant designs without manual adaptation. However, the biocompatibility, resorption, and mechanical behavior of sterilized PLDLLA/TCP for FOA need assessment, along with compatibility with common bone fixation systems like SonicWeld by KLS Martin.

Our study demonstrated that the robot-guided laser osteotomy grooves had a median deviation from the preoperative paths of approximately 0.44 mm, which is in a similar range as previously reported by Holzinger et al. [15] with the same system. In our study, a maximum deviation of 1.7 mm was observed, possibly due to registration errors from the camera navigation system or deformation of the plastic skulls under stress of the position marker clamp. Shape accuracy was assessed through surface scans (Fig. 6a, Table 2), showing a mean deviation of 0.04 mm for the 3D-printed skulls, with selective laser sintering possibly contributing to this error. The osteotomy deviation of the CARLO® is comparable to the linear AR-guided procedure described by García-Mato et al., who reported medial errors of < 1 mm for craniosynostosis osteotomies. Just like robot-guided procedures, AR seems to be able to provide accurate intraoperative guidance, providing real-time visual overlays of the VSP. AR systems are typically more accessible and less bulky than robot-guided systems; however, they rely on the surgeon’s manual precision to perform the osteotomy, introducing variability. Robot-guided systems offer automated execution, making them ideal for complex craniosynostosis procedures which require consistent accuracy. The integrated OCT system of the robot-guided osteotome enables precise depth control [26, 29]. However, considering the risk of neurological damage due to the proximity of meninges and brain tissue is crucial. The safety and accuracy of the depth measurement system of CARLO® primo +  requires further evidence through cadaver studies and pre-clinical trials.

One of the key challenges with navigation systems for pediatric cranial surgery is the difficulty to achieve rigid head fixation in infants with unfused sutures, open fontanelles and thin cranial bones, which also complicates the attachment of tracking reference tools for the navigation system. A potential solution for marker placement is the Modified Mayfield Rubber Stopper Technique, which employs soft rubber stoppers over Mayfield pins to evenly distribute pressure, thereby minimizing the risk of cranial injury [30] while providing a solid frame on which the optical markers are attached. This technique offers a less invasive approach to head stabilization, ensuring both safety and accurate tracking for navigation for pediatric cranial procedures.

The robot-guided laser was occasionally limited by the orbital cavity, necessitating manual cuts around this area which could have affected the overall accuracy of FOA. In the operating room, the robot’s large head and footprint, expenses, and the necessity to preserve delicate tissues prevent it from completely replacing conventional piezoelectric devices or drilling tools. In routine FOA procedures, incomplete osteotomies of the supraorbital bar are performed to achieve the desired cranial shape by bending the bone [31]. In these cases, the bone segments remain connected in non-expanded locations, allowing bending to the ideal position using the plate as a guide, improving stability between the bone segments. Likewise, instead of performing complete osteotomies, we propose that the pulse layers of the laser osteotomy could in future be reduced to merely indicate the cutting planes, enabling surgeons to complete osteotomies with conventional cutting devices, thereby reducing operational time. The osteotomy procedure lasted approx. three hours. Time is crucial in pediatric surgeries as longer procedures increase the risk of complications like blood loss and anesthesia-related issues. With improvements in navigation- and robotic systems and a better understanding of the needs of the patient and medical practitioners, we expect the operational time of the osteotomies to decrease, which will streamline the process and significantly reduce operative time, enhancing efficiency in future surgeries.

Despite various procedural complexities, the final median surface deviation of the FOA was relatively low, remaining within the range of 1.01–1.35 mm (Table 3). The maximum deviation of 3.51 mm around the orbital region likely resulted from the limited stability of the frontal plate, contributing to the medial rotation of bony segments lateral to the orbits. An in vivo median accuracy within this range is considered clinically acceptable, although, to our knowledge, no previous studies have quantitatively compared the accuracy of conventionally performed FOAs. Future research should investigate the impact plate design to optimally support repositioned bone segments.

Using the proposed workflow, the need for surgical template planning can be omitted, saving significant pre-operative planning time. Additionally, an efficient implant design reduces the number of screws needed, decreasing costs. We believe this technology will enhance the efficiency of surgical interventions, improve surgical outcomes, and reduce the risk of complications and revision surgeries.

## Conclusion

In this in vitro study, we have demonstrated a novel approach to performing FOA by efficiently transferring virtually planned data to the OR using the synergy of robot-guided laser osteotomy and patient-specific fixation plates. No surgical templates were required to precisely cut the bone and position the bony segments in the correct location. In future, further investigations must be performed to verify this workflow on bony tissue and prove the safety and effectiveness of this method.

## Data Availability

The datasets used and/or analyzed during the current study are available from the corresponding authors upon reasonable request.
